# Critical Analysis of PIM2 Score Applicability in a Tertiary Care PICU in Western India

**DOI:** 10.1155/2014/703942

**Published:** 2014-04-27

**Authors:** Vivek V. Shukla, Somashekhar M. Nimbalkar, Ajay G. Phatak, Jaishree D. Ganjiwale

**Affiliations:** ^1^Department of Pediatrics, Pramukhswami Medical College, Karamsad, Anand, Gujarat 388325, India; ^2^Central Research Services, Charutar Arogya Mandal, Karamsad, Anand, Gujarat 388325, India

## Abstract

*Objective. *Children have limited physiological reserve that deteriorates rapidly. Present study profiled patients admitted to PICU and determined PIM2 score applicability in Indian setting. *Patients and Methods. *Prospective observational study. *Results. *In 742 consecutive admissions, male : female ratio was 1.5 : 1, 35.6% patients were ventilated, observed mortality was 7%, and 26.4% were <1 year. The profile included septicemia and septic shock (29.6%), anemia (27.1%), pneumonia (19.6%), and meningitis and encephalitis (17.2%). For the first year, sensitivity of PIM2 was 65.8% and specificity was 71% for cutoff value at 1.9 by ROC curve analysis. The area under the curve was 0.724 (95% CI: 0.69, 0.76). This cutoff was validated for second year data yielding similar sensitivity (70.6%) and specificity (65%). Logistic regression analysis (LRA) over entire data revealed various variables independently associated with mortality along with PIM2 score. Another logistic model with same input variables except PIM2 yielded the same significant variables with Nagelkerke *R *square of 0.388 and correct classification of 78.5 revealing contribution of PIM2 in predicting mortality is meager. *Conclusion. *Infectious diseases were the commonest cause of PICU admission and mortality. PIM2 scoring did not explain the outcome adequately, suggesting need for recalibration. Following PALS/GEM guidelines was associated with better outcome.

## 1. Introduction

Infant and childhood mortality is very high in resource-limited countries. According to the 2010 United Nations reports, infant mortality rate (IMR) ranges from 1.92 (Singapore) to 135 (Afghanistan) with IMR of India at 52.9 deaths per 1000 live births [1]. About 60% of these deaths are neonatal yet many of the remaining deaths are preventable by appropriate and timely interventions. Mortality rates have declined in the last decade due to economic growth and better health care facilities, yet the rates still remain very high especially in rural areas [1]. The diseases accounting for the mortality also vary geographically.

Children have poor physiological reserve that deteriorates rapidly during life-threatening emergencies. Following evidence based guidelines for management of pediatric emergencies by implementing the Pediatric Advanced Life Support [2] guidelines (American Academy of Pediatrics) and the Golden Hour Emergency Management [3] of pediatric illnesses laid down by the Indian Academy of Pediatrics offers the possibility of a better outcome. Mortality and morbidity of pediatric illness depend on the rapidity of response and target oriented therapy [4].

For planning of future healthcare and emergency policies in pediatric population, it is imperative to understand the comprehensive profile of pediatric emergencies. Most of the studies depicting profile of patients presenting in emergency departments and those treated in PICU are either from the developed countries [5–12] or from the metropolitan cities of India [13–15]. Even continent-wide directories of profiles and outcomes of patients attending emergency care and admitted to pediatric intensive care have been prepared [16, 17].

There is paucity of data from rural and western part of India. Such data would be helpful in identification of the prevalent serious diseases likely to present at emergency department and also to form an early intervention strategy as well as prioritize and plan appropriate resource allocation. The present study is undertaken to assess the clinical profile of patients presenting to the emergency room of a rural, tertiary care center in western India, which will allow us to devise specific responses [9, 18, 19].

Illness severity and mortality risk scoring systems are used for predicting the outcome of children admitted to PICU [20]. These scoring systems cannot give individual risk very accurately but aid in comparing severity in patients with similar disease and presentation [21] for comparing the efficiency of different PICU [22, 23]. Many studies have validated the use of prognostic scores like PIM2, PRISM3, and so forth and their association with outcome of patients receiving intensive care in the west. Few such studies have been conducted in rural settings in the developing world. These areas account for large number of critical cases and global mortality burden. The most validated and widely studied score is pediatric index of mortality 2 score [24–27].

Shann et al. introduced pediatric index of mortality (PIM) in 1997 for prediction of outcome of patients admitted in PICUs of Australia, United Kingdom, and New Zealand [28, 29]. This system was updated in 2003 (PIM2) and is better than the previous version in outcome predictability [29]. A valid score should predict mortality with reasonable sensitivity and the variables used in calculating the score should be appropriate and in accordance with course of clinical management [30–32]. The main objective of our study was to assess the clinical profile of patients requiring pediatric intensive care and determine applicability of PIM2 score in a rural, tertiary care center in western India.

## 2. Methods

### 2.1. Study Setting

We conducted a prospective observational study covering patients admitted to pediatric (1 month to 18 years) intensive care units from January 2010 to December 2011. The study was approved and the human research ethics committee of the institute granted a waiver of informed consent.

### 2.2. Data Collection

The clinical and general profile variables were noted at the time of admission in PICU. All the profile data variables and observations were noted at the time of admission to PICU latest by the first 24 h after admission. Scoring was done with the laboratory investigations, which were clinically indicated in management of the patients. The interventions done at emergency care department and pediatric intensive care unit within the first 24 h of admission were recorded. The admission source, time taken to enter PICU from the point of admission, and interventions carried by emergency team and pediatric emergency team were noted. The interventions of both the emergency team at emergency department and pediatric emergency team at emergency department and at pediatric intensive care department for the first 24 h after admission were studied, and adherence to management of patients according to the PALS and GEM guidelines and protocol was assessed. Deviation from protocol was noted in every patient included in the study. All the intensive and life support interventions were noted. At each step the interventions were classified as appropriate or inappropriate according to the PALS and GEM guidelines. The outcome of the patients was recorded at the time of discharge. According to the diagnosis at the time of discharge the patients were classified according to the systems involved and major disease groups. Most of the patients who took discharge against medical advice (DAMA) did it for financial reasons in spite of the hospital policy to manage patients till alternative means are ensured. Further, most of them were in very critical condition and less likely to survive. Hence the DAMA patients were classified as Death for statistical analysis. The variable, namely, protein energy malnutrition, was removed from the analysis due to technical difficulties faced in weighing of critically ill patients with ongoing life support modalities.

### 2.3. Statistical Methods

The baseline data of the patients was expressed using descriptive statistics like mean, standard deviation, frequencies, proportions, and so forth. Various associations at the univariate level were expressed as cross tabulations and chi square statistics for qualitative variables. Independent sample *t*-test was used to express the associations for the continuous independent variables. The optimal cutoff value of PIM2 scores in this population was determined by ROC analysis. The independent contribution of various factors to mortality was determined using the multivariable stepwise logistic regression model with backward likelihood ratio (LR) method. The data was analyzed using SPSS 14.

## 3. Results

**Figure 1:**
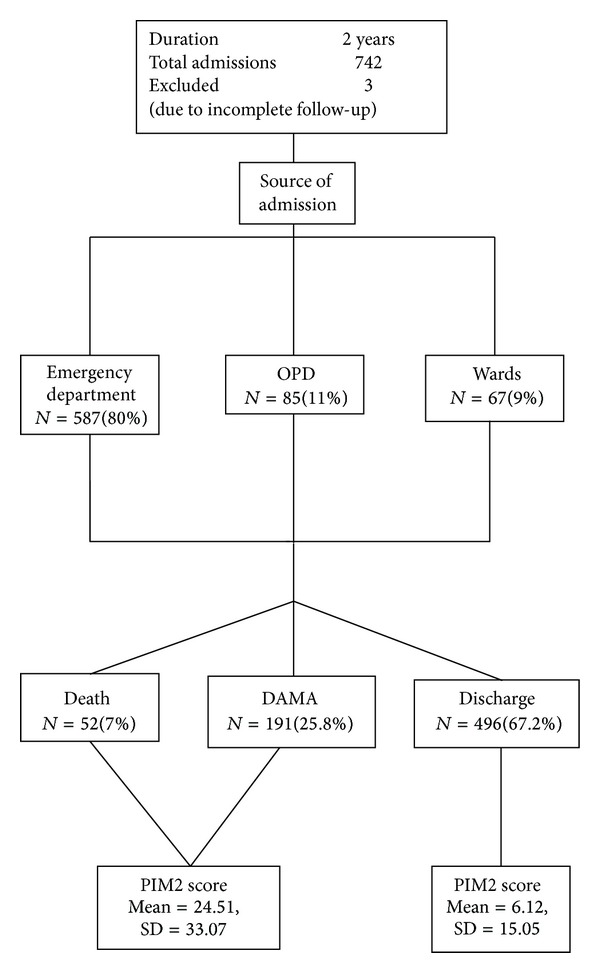
Flowchart of study participants. OPD: outpatient department, DAMA: discharge against medical advice, PIM2: pediatric index of mortality 2, and SD: standard.

A total of 742 patients were admitted to PICU during the study period (Figure 1). Three patients were excluded because of transfer to another hospital and incomplete follow-up. There was seasonal fluctuation in PICU admissions. The median age of patients was 36 months (range 1–216 months). Out of 742 patients included in study, 445 (60.2%) were males and 294 (39.8%) were females. Two hundred and sixty-one patients (35.3%) were mechanically ventilated during their PICU stay. Observed mortality was *N* = 52/739 (7%). After DAMA patients were considered dead, the mortality was *N* = 243/739 (32.8%). The most frequent diagnoses were clinically septicemia and septicemic shock 219 (29.6%), significant anemia *N* = 200 (27.1%), pneumonia *N* = 145 (19.6%), meningitis and encephalitis *N* = 127 (17.2%), multiple organ dysfunction *N* = 66 (8.9%), and congenital heart disease *N* = 42 (5.7%). The major causes of death were septicemic shock (*N* = 34, 65%), multiple organ dysfunction syndrome (*N* = 25, 48%), and meningitis/encephalitis (*N* = 11, 21.2%).

The numbers of patients with Death/DAMA were significantly more than those discharged in diagnoses subcategories of septicemic shock, multiple organ dysfunction, and meningitis/encephalitis.

The sensitivity of the PIM2 was found for the first year data as 65.8%, and the specificity was 71% for a cutoff value at 1.9 by the receiver operating characteristic curve (ROC) analysis. The area under the curve was 0.724 (95% CI: 0.69, 0.76). This cutoff was validated for the second year data, which yielded the similar sensitivity (70.6%) and specificity (65%). In univariate analysis, PIM2 score was observed to be significantly higher (*P* < 0.001) in patients who died (243) (mean = 24.51, SD = 33.07) as compared to those who survived (496) (mean = 6.12, SD = 15.05).

The logistic regression analysis (LRA) with backward likelihood ratio method was used to obtain independent relationship between the predictor variables and the mortality. The variables included in the model were age (completed years), gender, time to reach PICU (minutes), presence or absence of anemia, pneumonia, multiple organ dysfunction syndrome, meningitis, congenital heart disease, emergency team following guidelines, pediatric team following guidelines, volume administration, airway stabilization, oxygen administration, shock, ventilator therapy given, and PIM2. Results of LRA showed that the overall correct classification rate was good (79.7%). The Nagelkerke* R* square = 0.409 implies that 40.9% of the variation in the outcome variable is explained by the variables in the model.

Age, PIM2, meningitis/meningoencephalitis, multiple organ dysfunction syndrome, congenital heart disease, following PALS/GEM guidelines, and shock are significantly associated with mortality (Table 1).

Another logistic model with the same input variables except PIM2 in the model yielded the same significant variables at the end with Nagelkerke* R* square of 0.388 and correct classification of 78.5 implying that the contribution of PIM2 in predicting mortality is meager (data not shown).

## 4. Discussion

Eighty percent of total PICU admissions were through the emergency department, 11% were admitted from OPD, and 9% were admitted from the hospital wards. Majority were unplanned admissions. Similar trends are seen in Indian studies [14] but western studies and registries show that significant proportions (approx. 85%) of the total admissions in PICU are planned and postsurgical [17]. Thus emergency care services in India should be more prepared for handling critically ill children. This will require training the teams in implementing protocol-based management of patients rapidly.

*N* = 261 patients (35.3%) were mechanically ventilated during their PICU stay. *N* = 496/739 patients were discharged after completion of their treatment. Observed mortality was *N* = 52/739 (7%); the major causes of death were septicemic shock, multiple organ dysfunction syndrome, and meningitis/encephalitis. If DAMA patients were considered dead, the mortality was *N* = 243/739 (32.8%). In analysis of outcomes DAMA patients were considered dead as most of them were very critical at the time of DAMA. Most of DAMA cases were due to financial constraints which are particularly a very important factor in Indian settings due to lack of state run insurance coverage. During the course of the study insurance was not available, but subsequently there has been a move towards insurance for below poverty line families. This insurance, referred to as Rashtriya Swasthya Bima Yojna (RSBY), covers emergency care too. However many eligible patients do not carry RSBY cards as the process of getting enrolled is not known to many eligible families, and getting a card often involves corruption at the local self-government level. The mortality percentage is similar to other PICUs [1, 9, 14, 16, 17, 24]. Infectious diseases were the most common cause of PICU admission and mortality which is also seen in some recent studies [13–15, 18], in contrast to very low contribution of the same in western countries [16, 17, 24].

Higher numbers of males were admitted with a male: female ratio of 1.5 : 1 (445 versus 294). Even if we discount liberally for the skewed sex distribution in the study population, this difference in admission is stark. This difference is common in countries such as India which have a preference for male gender. However the possibility of males requiring more intensive care cannot be ruled out as similar results are also seen in studies performed in developed countries [16, 17, 25, 26].

Infants less than 1 year comprised 40% of total admissions. More than 50% of patients admitted were below 2 years of age. The median age of patients was 36 months (range 1–216 months). A higher vulnerability of infants and young children is present and a need for special consideration of these groups in healthcare planning is required. Similar results were also seen in other studies [9, 16–18].

Age group of age less than 2 years is associated with significantly higher disease frequency as well as higher chances of mortality and higher need for invasive life support. The association of pneumonia with age was significant (*P* < 0.0001). It was significantly higher in younger age groups and the disease frequency declined as the age increased. Significant associations with lower age group were noted in ventilatory requirements, meningitis/encephalitis, and septicemia/septic shock subgroups. Distribution of anemia and MODS was not correlating with age. Similar findings are also seen in other studies [17].

Analysis of monthwise frequency of different diseases showed a definite trend in disease frequency. Infectious diseases such as septicemia and septic shock, meningitis/encephalitis, and pneumonia had significant variation seasonally. Rainy season of June to August had significant increase in frequency of meningitis/encephalitis. Pneumonia frequency was seen more in winter months from August to December. Similar seasonal trends were also reported in other studies [16–18].

Only 1% of total patients were fully insured. The analysis showed that the outcome in insured patients was significantly better with no patients taking DAMA or dying as compared to uninsured groups. Patients (3%) having even a partial insurance (KAS) had a significantly better outcome. Category wise outcome analysis showed significantly better outcomes with patients having insurance (*P* value = 0.02).

The sensitivity (65.4%) and specificity (70.8%) of PIM2 were significantly lower in our study as compared to those in western studies [24–27]. These results show that PIM2 score is not well calibrated and not useful to predict mortality with low specificity and sensitivity, and recalibration of PIM2 score is needed according to Indian settings.

The stepwise logistic regression analysis (LRA) was used to obtain a risk adjusted relationship between the predictor variables and the probability of death. The LRA showed significant predictability of variables such as septicemic shock, meningitis/encephalitis, acute renal failure, and dialysis and ventilation during PICU stay with respect to the outcome which shows the need of incorporating these variables in prognostic mortality score in Indian setting.

Improvement of outcome on adherence to PALS and GEM guidelines indicates the need for proper implementation of guidelines for improving outcome in pediatric emergency care. The providers of emergency care to a child should have appropriate and proper training in managing critical children according to PALS/GEM guidelines. Similar results were also seen in another study [4].

### 4.1. PIM2 Applicability in Indian Settings

The PIM2 score was calibrated to predict mortality with a level of healthcare facilities available in Australia, New Zealand, and United Kingdom at the time of study, that is, 1997 around 16 years back so it cannot be generalized without proper recalibration. The study population is 1 month to 18 years as compared to original study of PIM2 including the patients <16 years of age by Lemeshow and Gall [30]. The score validity would differ with changes in treatment and management approaches, level of healthcare, referral practices, and admission criteria [29]. The high risk and low risk diagnosis in PIM2 were analyzed according to those of Australia, New Zealand, and United Kingdom [29], none of which are suitable for southeast Asian countries including India.

The laboratory parameters and ventilation during the first hour of admission which are significant variables in calculating PIM2 are subjective to availability of laboratory tests, treatment approaches, and intervention thresholds which changes the mortality score calculation even with same disease severity [29]. Many patients admitted in tertiary level Indian PICU are referred from local hospitals. These patients are stabilized before referrals which alters the parameters included in calculation of score. PIM2 does not take into account the referred status and the treatment received; this hampers the assessment of actual risk of mortality.

It can only compare groups but cannot predict risk in individual patients [29]. Normal systolic blood pressure is taken as 120 mmHg whereas most of pediatric patients have much less than 120 mmHg.

Age is not taken into account while calculating risk in PIM2 score when it is well evident that young children have higher vulnerability. The low risk diagnoses such as croup, bronchiolitis, asthma, and diabetic ketoacidosis have significant mortality in a resource-limited country like India.

The high risk diagnoses which are included in PIM2 such as severe combined immunodeficiency, leukemia, neurodegenerative disorders, spontaneous cerebral hemorrhage, and hypoplastic left heart are infrequent causes of mortality in India, whereas infectious diseases, septicemia, septicemic shock, meningitis and encephalitis, multiple organ dysfunction syndrome, acute renal failure, and disseminated intravascular coagulation which account for majority of deaths in developing countries are not considered in calculation of PIM2 scoring [30]. These lacunae make PIM2 score inappropriate for use in Indian setting without proper recalibration.

## 5. Conclusion

PIM2 score is not applicable without proper recalibration in Indian settings where the disease patterns and frequency are markedly different and the standard of care provided is not as good as that provided in developed countries where PIM2 was devised and validated.

In our setup there is delay in seeking health and most patients end up in ICU because of late care received. This contributes to severe physiologic derangements and increased chances of mortality which is in contrast to western and developed countries due to availability of good referral and immediate healthcare assistance. There is need for proper intervention and management at the primary centers and well-structured referral system.

## 6. Implications

### 6.1. Generalizability

This study done in Indian settings may also be applicable to other developing and underdeveloped countries of the world.

### 6.2. Strengths and Limitations

The study has been carried out over a fairly large period and can avoid the changes in results due to differences in seasons and patterns of admissions. Though it is a single center study, being a referral center it receives most of the sick patients in the region due to a large referral. This study is the only study till date from India to validate PIM2 score, which is validated across many developed countries and a few developing countries.

Due to financial constraints many patients took DAMA and this might result in variations in outcome when reproduced in a more affluent setting. We did not collect data related to prematurity in the study population as often this data is not available and may be unreliable as patients may have been delivered at home or delivered by trained birth attendants who cannot assess prematurity. Our patient population extended until 18 years and collecting this data over the entire population would have been challenging.

### 6.3. Further Research

Further research is needed to confirm the findings of present study in a multicentric study from areas of developing countries. There is a need to have risk scoring calibrated to local disease prevalence, treatment expertise, malnutrition status, and other factors contributing to mortality.

There cannot be a compact risk scoring system because the smaller the variable size the lesser the sensitivity and specificity. So there is a need of mortality predictor score with sufficient variables which takes into account wider range of medical problems and possible contributors to mortality, without having much emphasis on laboratory parameters, and which does not get biased by different thresholds of interventions.

Many diseases occurring in tropical countries, which account for significant mortality, evolve over a period of time and the prediction of outcome at the admission time is flawed largely in such patients. This denotes a need for a score, which continuously estimates the risk over a period.

## What Is Known on This Subject

PIM2 score is a validated and widely used mortality prediction score in pediatric critical care. It has been proved to be most accurate in mortality prediction in developed country setting.

## What This Study Adds

PIM2 is a poor mortality prediction model for developing country settings due to resource-poor conditions and difference in disease prevalence. This study highlights factors associated with mortality in these settings that can be considered for mortality prediction models.

## Figures and Tables

**Table 1:** Findings of logistic regression analysis with PIM2 as a predictor in the model.

Variable	Categories	OR	*P* value	95% CI for OR
PIM2	Continuous	1.017	0.0001	1.007, 1.026
Age (completed years)	Continuous	0.955	0.028	0.917, 0.995
MODS	Yes (ref)			
	No	0.161	0.001	0.062, 0.417
Meningitis	Yes (ref)			
	No	0.239	0.001	0.145, 0.395
CHD	Yes (ref)			
	No	0.396	0.035	0.167, 0.936
PALS/GEM guidelines followed	Yes (ref)			
	No	6.351	0.0001	3.008, 13.412
Shock	Yes (ref)			
	No	0.325	0.001	0.205, 0.513

PIM2: pediatric index of mortality 2, OR: odds ratio, CI: confidence interval, MODS: multiple organ dysfunction syndrome, and CHD: congenital heart disease.

## Abbreviations

DAMA: Discharge against medical advice
LRA: Logistic regression analysis
MODS: Multiple organ dysfunction syndrome
PIM2: Pediatric index of mortality 2
PICU: Pediatric intensive care unit
ROC: Receiver operating characteristic curve.
